# Mating Types of *Ustilago esculenta* Infecting *Zizania latifolia* Cultivars in Japan Are Biased towards MAT-2 and MAT-3

**DOI:** 10.1264/jsme2.ME23034

**Published:** 2023-09-14

**Authors:** Yuka Chigira, Nobumitsu Sasaki, Ken Komatsu, Kouji Mashimo, Shigeyuki Tanaka, Minori Numamoto, Hiromitsu Moriyama, Takashi Motobayashi

**Affiliations:** 1 Graduate School of Agricultural Science, Tokyo University of Agriculture and Technology, Fuchu, Tokyo 183-8509, Japan; 2 Gene Research Center, Tokyo University of Agriculture and Technology, Fuchu, Tokyo 183-8509, Japan; 3 Institute of Global Innovation Research (GIR), Tokyo University of Agriculture and Technology, Fuchu, Tokyo 183-8509, Japan; 4 Field Science Center, Tokyo University of Agriculture and Technology, Fuchu, Tokyo 183-8509, Japan; 5 Faculty of Agriculture, Setsunan University, Hirakata, Osaka 573-0101, Japan

**Keywords:** *Ustilago esculenta*, *Zizania latifolia*, makomotake, mating type, haploid isolation

## Abstract

*Zizania latifolia* cultivars infected by the endophytic fungus *Ustilago esculenta* develop an edible stem gall. Stem gall development varies among cultivars and individuals and may be affected by the strain of *U. esculenta*. To isolate haploids from two *Z. latifolia* cultivars in our paddy fields, Shirakawa and Ittenkou, we herein performed the sporadic isolation of *U. esculenta* strains from stem gall tissue, a PCR-based assessment of the mating type, and *in vitro* mating experiments. As a result, we obtained heterogametic strains of MAT-2 and MAT-3 as well as MAT-2, but not MAT-3, haploid strains. Another isolation method, in which we examined poorly growing small clusters of sporidia derived from teliospores, succeeded in isolating a MAT-3 haploid strain. We also identified the mating types of 10 *U. esculenta* strains collected as genetic resources from different areas in Japan. All strains, except for one MAT-1 haploid strain, were classified as MAT-2 haploid strains or heterogametic strains of MAT-2 and MAT-3. The isolated strains of MAT-1, MAT-2, and MAT-3 mated with each other to produce hyphae. Collectively, these results indicate that the mating types of *U. esculenta* infecting *Z. latifolia* cultivars in Japan are biased towards MAT-2 and MAT-3 and that *U. esculenta* populations in these Japanese cultivars may be characterized by the low isolation efficiency of the MAT-3 haploid.

*Zizania latifolia*, known as Manchurian wild rice, is a perennial aquatic grass belonging to the genus *Zizania* in the family Gramineae and is distributed in East and Southeast Asia, including Japan. In agriculture, *Z. latifolia* is cultivated as a vegetable in many Asian countries because its varieties infected by the endophytic fungus *Ustilago esculenta* develop an edible stem gall, known as “jiaobai” in China (Zhang *et al.*, 2017), “kamborg” in India (Jose *et al.*, 2016), and “makomotake” in Japan (Kawagishi *et al.*, 2006). Infection by this smut fungus prevents inflorescence development and seed production by the plant. Instead, the hyphae and mycelia of the fungus spread throughout culm tissue near the apical meristem of young culms, causing a swollen stem gall (Yang and Leu, 1978; Zhang *et al.*, 2012). During gall formation, large hyphal aggregates produce the sori forming mature teliospores (Yang and Leu, 1978). White stem galls with less sori are edible and a more valuable vegetable than gray-to-brown galls full of sori. The efficiency of sorus formation varies depending on the strain of *U. esculenta*.

A mycelial-teliospore (M-T) strain and teliospore (T) strain of *U. esculenta* have been isolated and characterized in detail in China (Yang and Leu, 1978; You *et al.*, 2011, Zhang *et al.*, 2017; Liang *et al.*, 2019). The infection of *Z. latifolia* with M-T strains results in the formation of edible white stem galls (called white jiaobai) because no sori are formed at the harvesting stage. On the other hand, T strains develop sori at the early stage of stem gall formation. Mature stem galls filled with sori (called gray jiaobai) are not edible. M-T strains show lower rates of teliospore germination and hyphal growth, require different nutrients in medium for growth, and are more sensitive to external signals, such as H_2_O_2_ and pH, than T strains (Zhang *et al.*, 2017). In addition, M-T strains, which are more endophytic than T strains, have multiple mutated genes related to pathogenicity (Zhang *et al.*, 2017).

*U. esculenta* is a bipolar heterothallic fungus with three mating-type loci: MAT-1, MAT-2, and MAT-3 (Liang *et al.*, 2019). Sporidia that develop from the teliospore multiply asexually through repeated budding. Each sporidium is a haploid carrying the MAT-1, MAT-2, or MAT-3 locus. Mating occurs when two conjugation tubes of haploids with different mating types fuse together, producing dikaryotic hyphae. By characterizing the mating types of M-T and T strains, the MAT locus-related genes involved in the initial step of mating between sexually compatible strains and the later steps of hyphal growth and invasion after mating have been identified (Zhang *et al.*, 2019), such as the *a* locus genes for pheromones (*mfa*) and pheromone receptors (*pra*) and the *b* locus genes for homeodomain proteins (*bE* and *bW*). The mfa pheromone and pra receptor proteins are necessary for the recognition of compatible haploid sporidia (Kellner *et al.*, 2011). bE and bW are subunits of the heterodimeric transcription factor responsible for hyphal formation, elongation, and invasion after mating (Wahl *et al.*, 2010).

In Japan, *Z. latifolia* cultivars producing edible makomotake are expected to be cultivated in fallow rice fields (Anazawa *et al.*, 2007) for the following reasons: (1) there is no need to invest in new agricultural machinery because machines for rice fields may be used, (2) its planting and harvesting seasons are different from those of rice paddy fields, and (3) its economic value is higher than that of rice cultivation. *Z. Latifolia* cultivars in Japan are divided into two types: early maturing cultivars, such as Ittenkou, which form the stem gall from mid-September, and late maturing cultivars, such as Shirakawa, which form the stem gall later. Regardless of the type of cultivar, we often find individual plants that develop the stem gall much later than the harvest season and call these the “delayed type” to distinguish them from the “normal type”. These variations in the timing of stem gall formation result in the inefficient production of makomotake. To improve the productivity of makomotake, it is important to identify the cause of variations in the timing of stem gall formation between individual plants. As described above, the different characteristics of *U. esculenta* strains may have different effects on the development of the stem gall. However, limited information is currently available on *U. esculenta* strains infecting Japanese *Z. latifolia* cultivars used for the production of edible makomotake. Therefore, the isolation of *U. esculenta* haploid strains that infect Japanese *Z. latifolia* cultivars and the establishment of an experimental infection system using mating pairs between isolated haploids are needed. We herein obtained haploid strains of *U. esculenta* from *Z. latifolia* cultivated in our paddy fields and in different areas of Japan and identified their mating types by genomic PCR and *in vitro* mating experiments.

## Materials and Methods

### Isolation of *U. esculenta* strains from the stem gall tissue of *Z. latifolia*

*Z. latifolia* plants with the stem gall (cvs. Ittenkou and Shirakawa; Supplementary Fig. S1), which were cultivated in 2019 at the paddy fields of Tokyo University of Agriculture and Technology (Honmachi, Fuchu, Tokyo), were collected. After the leaf sheaths around the stem gall were removed, the surface of the stem gall was wiped with 70% ethanol. Internal stem tissue was cut out‍ ‍on a clean bench using flame-sterilized tweezers and razor blades. Excised tissues were incubated on potato dextrose agar (PDA) containing chloramphenicol (0.05‍ ‍g‍ ‍L^–1^) and streptomycin (0.1‍ ‍g‍ ‍L^–1^) at 28°C for 10–15 days. Colonies that formed around the excised tissues on PDA were transferred onto new PDA and incubated at 28°C. Each colony was suspended in sterile RO water, placed on PDA, and incubated at 28°C. Well-grown single colonies were saved individually and used for characterization. To examine the formation of aerial hyphae, colonies were transferred to YEPS agar medium containing sucrose (20‍ ‍g‍ ‍L^–1^), tryptone (20‍ ‍g‍ ‍L^–1^), yeast extract (10‍ ‍g‍ ‍L^–1^), activated charcoal (10‍ ‍g‍ ‍L^–1^), and agar (15‍ ‍g‍ ‍L^–1^) and incubated at 28°C.

### *In vitro* mating experiment

Strains of *U. esculenta* from yeast-like colonies were cultured separately on YEPS agar medium at 28°C. Clumps of each strain that formed on the medium were scraped with a sterile toothpick and resuspended in YEPS liquid medium to an OD_600_ of approximately 1.5. In *in vitro* mating experiments, pairs of selected strains were mixed at a ratio of 1:1, and approximately 30‍ ‍μL of the mixture was dropped onto YEPS agar medium. YEPS agar media were kept at 28°C for more than 2‍ ‍weeks.

### DNA isolation and PCR

DNA isolation and PCR were performed to confirm that the isolated strains were *U. esculenta* and identify their mating types. Isolates were cultured on YEPS agar or PDA medium and a clump of approximately 3‍ ‍mm in diameter was scraped from each medium with a disposable inoculation loop and collected in a 2.0-mL tube. These tubes were frozen at –30°C until used. Six hundred microliters of TE with 50% (w/v) Chelex-100 (Bio-Rad Laboratories) and 200‍ ‍μL of chloroform were added to the fungal clumps. Clumps were homogenized with a Mini Bead-Beater (WakenBtech) at 4,800‍ ‍rpm for 1‍ ‍min. After centrifugation at 20,400×*g* for 10‍ ‍min, 150‍ ‍μL of the supernatant was collected and an equal volume of chloroform was added and vortexed well. After centrifugation at 20,400×*g* for 5‍ ‍min, 100‍ ‍μL of the supernatant was used as the DNA extract.

Genomic PCR was performed using the Ex Taq Hot Start Version kit (TaKaRa Bio), the primers listed in Table 1, and TaKaRa PCR Thermal Cycler Dice (TaKaRa Bio). Thirty cycles of PCR were conducted under the following conditions according to the manufacturer’s instructions: denaturation at 98°C for 10‍ ‍s, annealing at 50°C for 30‍ ‍s, and elongation at 72°C for 50 s. PCR products were electrophoresed on 1% agarose gel with TAE buffer, stained with ethidium bromide, and visualized with a Gel-Doc RX+ imaging system (Bio-Rad Laboratories).

### Isolation of the MAT-3 haploid strain from *U. esculenta* teliospores

Teliospores in the stem gall of a *Z. latifolia* plant cultivated in 2021 were scraped off with an autoclaved toothpick, suspended in 100‍ ‍μL of sterile RO water to make a teliospore suspension, and then diluted 10-fold with sterile RO water. Ten microliters of the diluted suspension was dropped onto the periphery of PDA medium and flowed in a straight line with the medium tilted. Six plates of PDA medium were prepared in the same manner. After being incubated at 28°C for 6 days, 107 small colonies were picked up with the tip of an ethanol-sterilized sewing needle under an Olympus SZX9 stereomicroscope (Olympus) and transferred onto the same PDA and new YEPS agar (without active charcoal) media at intervals of approximately 1‍ ‍cm. After being incubated at 28°C for 7 or 8 days, the diameter of each colony was measured with a ruler or, if colony formation was invisible, the growth of sporidia was examined under the stereomicroscope. Strains with a maximum diameter ≤2‍ ‍mm and without mycelial growth were selected for the mating-type ana­lysis. Visually confirmed colonies were scraped off and inoculated into YEPS (without active charcoal) liquid medium. When strains growing sporidia were confirmed by the stereomicroscope only, their colonies were cut out together with agar medium and inoculated to YEPS (without active charcoal) liquid medium. YEPS liquid media were incubated at 28°C and 180‍ ‍rpm with reciprocal shaking for 18 days (strains A12, A13, α20, α25, β11, and γ17) or 48 days (α1, β3, β8, and β9).

### *U. esculenta* strains from the Genebank of the National Agriculture and Food Research Organization (NARO) Research Center of Genetic Resources

Strains of *U. esculenta* isolated from *Z. latifolia* plants produced in various areas of Japan (Nagano, Mie, Shiga, Okinawa, and Ibaraki) and China (the specific production area is unknown) were obtained from the NARO Genebank, the Research Center of Genetic Resources (https://www.gene.affrc.go.jp/index_en.php). MAFF numbers, strain names, and production areas are summarized in Table 4; however, the specific names of *Z. latifolia* cultivars are unavailable. Cultivation on PDA medium, mating assays, and genomic PCR of the strains were performed as described above.

### Microscope observations

**Fig. 1.**
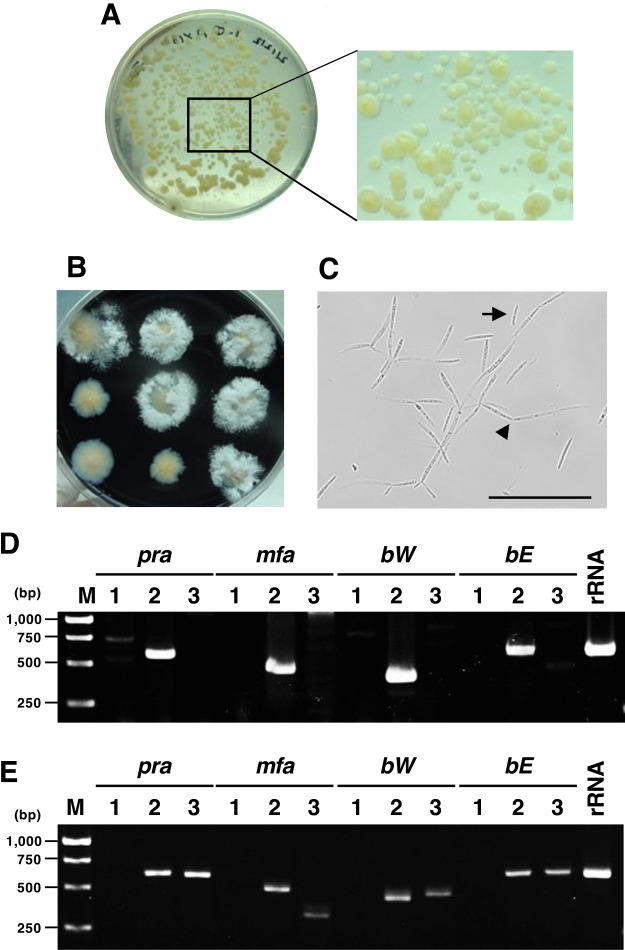
Colony formation by *Ustilago esculenta* on PDA and YEPS agar media and PCR-based mating-type assays. A and B. Yeast-like colonies of *U. esculenta* on PDA medium after an incubation at 28°C for 39 days (A). There were colonies of various sizes. These yeast-like colonies consisted of a *U. esculenta* population, which was isolated from the stem gall tissue of *Zizania latifolia*. Colonies of each strain with or without aerial hyphae cultured on YEPS agar medium, incubated at 28°C for 35 days after transfer from the PDA medium (B). C. We observed pseudohyphae (arrowhead) and sporidia (arrow) from a colony without aerial hyphae cultured on YEPS agar medium at 28°C for 13 days (bar=100‍ ‍μm). D and E. Genomic PCR was performed with DNA samples from a colony without (D) or with (E) aerial hyphae. In D, the PCR products of all MAT-2-associated genes (*pra2*, *mfa2*, *bW2*, and *bE2*) were amplified, while non-specific fragments of unexpected sizes were weakly amplified for other genes. In E, the PCR products of all MAT-2- and MAT-3-associated genes (*pra2*, *pra3*, *mfa2*, *mfa3*, *bW2*, *bW3*, *bE2*, and *bE3*) were amplified. In both samples, the PCR products of the precursor rRNA gene (rRNA) of *U. esculenta* was amplified. The expected sizes of PCR products are listed in Table 1.

**Fig. 2.**
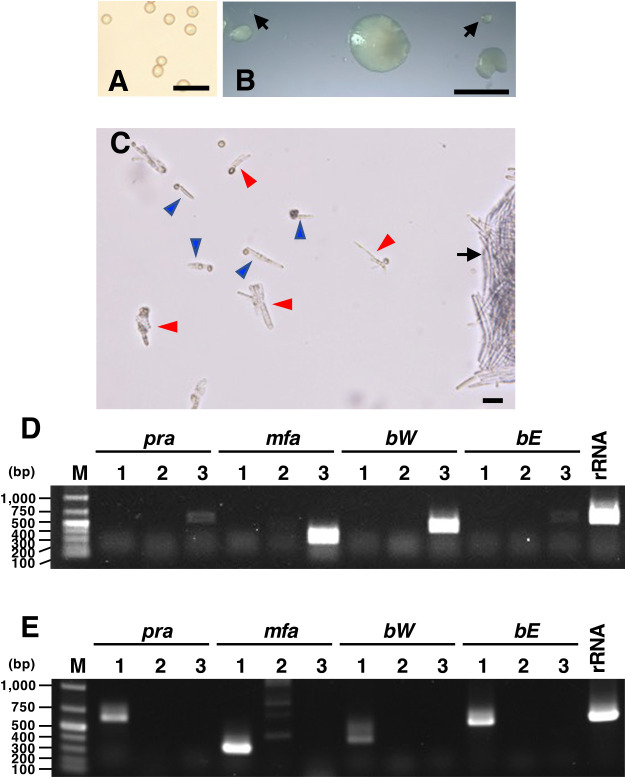
Isolation of MAT-3 and MAT-1 haploid strains of *Ustilago esculenta*. A. Teliospores from the stem gall of *Zizania latifolia* cv. Shirakawa. Bar=20‍ ‍μm. B. Colonies from teliospores on PDA medium after an incubation at 28°C for 6 days. Arrows indicate the colonies selected for the isolation of MAT3 haplotype strains. Bar=2‍ ‍mm. C. Teliospores that ceased hyphal growth on PDA medium after an incubation at 28°C for 5 days. The arrow indicates a colony that continued to grow. Some teliospores did not develop sporidia from the promycelium (blue arrowheads) and others appeared to have stopped forming sporidia sometime after budding (red arrowheads). Bar=20‍ ‍μm. D and E. Mating-type assays of the MAT-3 (D) and MAT-1 (E) haploid strains by genomic PCR. The PCR products of MAT-3-associated genes (*pra3*, *mfa3*, *bW3*, and *bE3*) and MAT-1-associated genes (*pra1*, *mfa1*, *bW1*, and *bE1*) were amplified specifically for strain α1 and strain MJ-2, respectively. The precursor rRNA gene (rRNA) of *Ustilago esculenta* was amplified for both strains. The expected sizes of PCR products are listed in Table 1.

**Fig. 3.**
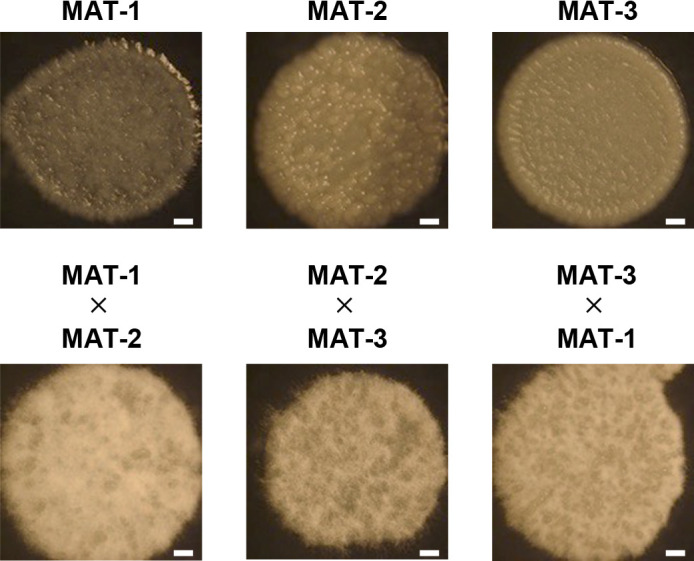
Morphology of colonies formed in the *in vitro* mating experiment using three different mating-type strains. Sporidia of three strains alone (MAT-1, MAT-2, and MAT-3) or a combination of two strains (MAT-1×MAT-2, MAT-2×MAT-3, and MAT-3×MAT-1) were grown on YEPS agar medium at 28°C. Images were captured after a 14-day incubation. MAT-1=strain MJ-2; MAT-2=strain Sn-1-(1)-F1; MAT-3=strain α1. Bar=1‍ ‍mm.

Yeast-like colonies, sporidia, mycelia, and teliospores were observed with an Olympus SZX9 stereomicroscope (Olympus) (for Fig. 1A, 2B and C, and 3), a Sony Cyber-shot DSC-HX5 camera (Sony) (for Fig. 1B), an Olympus BX50 biological microscope (Olympus) (for Fig. 2A), and a Keyence BZ-X800 all-in-one fluorescence microscope (Keyence) (for Fig. 1C).

## Results

### Isolation of haploid strains of *U. esculenta* from the stem gall tissue of *Z. latifolia*

To characterize *U. esculenta* infecting *Z. latifolia* plants (cvs. Ittenkou and Shirakawa) grown in our paddy fields, we attempted to isolate haploid strains from the stem gall: four stem galls from Ittenkou (I-1 to I-4 for the normal type only) and four stem galls from Shirakawa plants (two normal types named Sn-1 and Sn-2 and two delayed types named Sd-2 and Sd-3). We selected whitish yeast-like colonies on PDA medium, which were previously shown to be characteristic of *U. esculenta* (Zhang *et al.*, 2020). These original colonies were numbered in parentheses (*e.g.*, I-1-(1)) when obtained from different parts of the same stem gall (Table 2). Suspensions of these original colonies were repeatedly transferred to PDA medium. Among colonies of various sizes grown on PDA medium (Fig. 1A), well-grown single colonies were selected for transfer to YEPS agar medium containing activated charcoal, which promotes hyphal growth by mating (Rodríguez-Kessler *et al.*, 2012). Through attempts to isolate haploid strains, we found that there were colonies with or without aerial hyphae (Fig. 1B). Regarding the colony with aerial hyphae where two haploid strains may be mixed, approximately 20 days (for Shirakawa) or 15 days (for Ittenkou) were generally needed before aerial hyphae were observed visually on the colony. Isolated strains selected from several colonies were named with letters (*e.g.*, I-1-(1)-A). In addition, to completely isolate haploid cells, we obtained passage cultures of Sn-1-(1)-B and F, which showed aerial hyphal formation. As a result, progeny strains that did not form aerial hyphae in the colony were successfully isolated (*i.e.*, Sn-1-(1)-B2b, F1, and F2) (Table 2). These colonies without aerial hyphae formed pseudohyphae containing aerial cell chains and producing sporidia (Fig. 1C).

To identify haploid mating pairs, *in vitro* mating experiments were performed with 18 putative haploid strains that did not form colonies with aerial hyphae (13 from Shirakawa and 5 from Ittenkou) (Table 2). However, all of the crossings between the selected haploid strains resulted in the formation of yeast-like colonies that never developed aerial hyphae (data not shown). This result suggests that all the haploid strains used for crossing had the same mating type. To identify the mating type of these haploid strains, genomic PCR was performed using MAT-associated genes (*pra*, *mfa*, *bW*, and *bE*) (Table 1). PCR results showed that all of the haploid strains contained *pra*, *mfa*, *bW*, and *bE* genes associated with MAT-2, but not MAT-1 or MAT-3 (Fig. 1D and Table 2). This result clearly indicated that all strains used for *in vitro* mating experiments were MAT-2. In addition, we performed genomic PCR for 10 other strains that formed colonies with aerial hyphae (5 each from Shirakawa and Ittenkou) to clarify their mating types. As a result, all of the non-haploid strains contained the genes associated with MAT-2 and MAT-3, but not MAT-1 (Fig. 1E and Table 2). These results indicate that the two cultivars, Ittenkou and Shirakawa, were infected with the *U. esculenta* population of MAT-2 and MAT-3 and also that MAT-2 haploids were more preferentially isolated than MAT-3 haploids.

### Isolation of the MAT-3 haploid strain from poorly growing colonies from *U. esculenta* teliospores

Since the mating type of the haploid strains derived from well-grown colonies was only MAT-2, we hypothesized that the growth of MAT-3 haploid colonies was markedly slower than that of MAT-2 haploid colonies, resulting in MAT-3 haploid strains not being isolated. To obtain MAT-3 haploid strains, we observed colonies of the early progeny sporidia budded from teliospores that were collected from the stem gall of Shirakawa (normal type) (Fig. 2A). After an incubation for 6 days, we found a number of small colonies (sporidia clusters) of less than 0.5‍ ‍mm in diameter (Fig. 2B). Moreover, many teliospores developed no or a few sporidia from the promycelium (Fig. 2C), which is consistent with previous findings (You *et al.*, 2011; Zhang *et al.*, 2017). Among the 107 small colonies examined for colony formation, we obtained 10 strains that formed colonies or sporidial clusters of less than 2‍ ‍mm in diameter after an incubation for 7 to 8 days (Table 3). The amplification of the mating type-related genes of these 10 strains by genomic PCR revealed one haploid strain (named α1) of MAT-3, 5 haploid strains of MAT-2, and 4 heterogametic strains of MAT-2 and MAT-3 (Fig. 2D and Table 3). We accidentally detected the growth of strain α1 after a 48-days incubation, which was markedly longer than the time required for the growth of many other strains (18 days) (see Materials and Methods). These results support our hypothesis that MAT-3 haploid strains of *U. esculenta* in Shirakawa may grow more slowly than MAT-2 haploid strains.

### Identification of mating types of *U. esculenta* strains collected from different areas in Japan

To investigate whether the predominance of MAT-2 and MAT-3 was limited to Shirakawa and Ittenkou, we also obtained 10 strains of *U. esculenta*, which are genetic resources collected from different areas of Japan (*i.e.*, Nagano, Mie, Shiga, Okinawa, and Ibaraki Prefectures), and performed genomic PCR to investigate their mating types. One available strain from China was included in this experiment. As shown in Table 4, five strains from Nagano (TY64 and TY65), Mie (MC-2), Okinawa (ON-3), and Ibaraki (J5) were the MAT-2 haploid, while the remaining four strains from Okinawa (IF-6), Ibaraki (J1 and J8), and China (yeast4) were heterogametic strains of MAT-2 and MAT-3. Only one strain from Shiga (MJ-2) was the MAT-1 haploid (Fig. 2E and Table 4). These results suggest that Japanese cultivars of *Z. latifolia* are predominantly infected by the *U. esculenta* population with MAT-2 and MAT-3 mating types.

*In vitro* mating experiments using pairs of different mating types were conducted to confirm their mating ability. Strain MJ-2 and strain α1 were used as MAT-1 and MAT-3 haploid strains, respectively. One of the strains from Shirakawa, named Sn-1-(1)-F1, was used as the representative MAT-2 haploid strain. Sporidia of three strains (MAT-1, MAT-2, and MAT-3) alone formed yeast-like colonies (Fig. 3). On the other hand, the mating of two different strains resulted in the formation of colonies with aerial hyphae in‍ ‍all cases (MAT-1×MAT-2, MAT-2×MAT-3, and MAT-3×MAT-1) (Fig. 3). We also performed *in vitro* mating experiments using the MAT-2 haploid strain from Ittenkou, I-2-(3)-A, instead of Sn-1-(1)-F1. The results obtained confirmed that any combination of three different mating type strains led to hyphal development in their colonies (Supplementary Fig. S2). These results demonstrated the validity of our genomic PCR to identify haploid mating types.

## Discussion

In the present study, we focused on isolating *U. esculenta* haploid strains from Ittenkou and Shirakawa cultivars in our paddy fields. The isolation of sporidia using stem gall tissue provided colonies with or without hyphal growth. Analyses to identify mating types revealed that the *U. esculenta* population in the two cultivars consisted of the heterogametic strains of MAT-2 and MAT-3, excluding MAT-1, regardless of the cultivars or the normal or delayed phenotypes. All strains from yeast-like colonies without hyphal formation were the MAT-2 mating type. This result indicates that MAT-2 haploids were easily isolated from normally growing colonies, while MAT-3 haploids were more difficult to be isolated in the same manner. Our attempt to isolate MAT-3 haploids from teliospore-derived, poorly growing colonies led to the isolation of strain α1. Except for strain α1, only MAT-2 haploid strains were isolated from tiny visible colonies. This result suggests that the sporidial growth of MAT-3 was slower than that of MAT-2 or ceased shortly after germination from the teliospore. Consequently, slow-growing MAT-3 haploids may have been buried by fast-growing MAT-2 haploids during colony formation. Although further ana­lyses of the growth of MAT-3 haploid strains are required, the different characteristics of sporidial growth between the two haploid types may explain why haploid isolation from stem gall tissue resulted in the occupation of only MAT-2 strains. Therefore, a more careful examination of small colonies is needed for the isolation of MAT-3 haploid strains.

We also identified the mating types of 10 strains of *U. esculenta* collected from various regions of Japan. Consistent with Shirakawa and Ittenkou, MAT-2 alone or the combination of MAT-2 and MAT-3 was detected in all strains, except for strain MJ-2. These results suggest that the combination of MAT-2 and MAT-3 is the predominant mating type of the *U. esculenta* population infecting *Z. latifolia* cultivars in Japan. In addition, no haploid strains of MAT-3 were identified from the *U. esculenta* collection, which provides support for the difficulties associated with isolating MAT-3 haploids from Japanese cultivars of *Z. latifolia*. Regarding the MJ-2 strain, which was the only MAT-1 haploid identified, its source was *Z. latifolia* with a blackish brown stem gall full of teliospores (Supplementary Fig. S3). Pure teliospores are used as a pigment called “makomozumi” in the lacquer industry in Japan, but may be a cause of hypersensitivity pneumonitis (Yoshida *et al.*, 1996; Fujii *et al.*, 2007). Therefore, the *Z. latifolia* cultivar carrying the MJ-2 strain may be for craft (“makomozumi” pigment use) rather than for food. Zhang *et al.* (2017) reported that the mating types of T strains from gray jiaobai, which is full of sori, were MAT-1 or MAT-2, while those of the M-T strain from white jiaobai were MAT-2 or MAT-3. Based on these findings, MAT-1 strains rather than MAT-2 and MAT-3 strains may play a dominant role in facilitating the development of teliospores and sori in the stem gall. On the other hand, the infection of *Z. latifolia* by MAT-2 and MAT-3 strains may be responsible for the M-T phenotype, which produces white edible galls with no or few sori.

In conclusion, the present results indicate that Japanese cultivars of *Z. latifolia* are infected by the *U. esculenta* strains of MAT-2 and MAT-3 mating types. To further clarify the relationship between stem gall formation and *U. esculenta* strains, it is important to isolate more MAT-3 strains from Shirakawa, Ittenkou, and other cultivars. As previously reported (Liang *et al.*, 2019), the isolation of sporidia immediately after budding from teliospores using a micromanipulator is an effective method to isolate MAT-3 strains. Further characterization and comparative genomic ana­lyses of MAT-2 and MAT-3 strains infecting *Z. latifolia* cultivars in Japan will provide more information on the characteristics of *U. esculenta*, which will improve the productivity of makomotake.

## Citation

Chigira, Y., Sasaki, N., Komatsu, K., Mashimo, K., Tanaka, S., Numamoto, M., et al. (2023) Mating Types of *Ustilago esculenta* Infecting *Zizania latifolia* Cultivars in Japan Are Biased towards MAT-2 and MAT-3. *Microbes Environ ***38**: ME23034.

https://doi.org/10.1264/jsme2.ME23034

## Supplementary Material

Supplementary Material

## Figures and Tables

**Table 1.** Primers used in this study^1^

Gene	Forward	Forward primer (5'→3')	Reverse	Reverse primer (5'→3')	Expected DNA size (bp)
*pra1*	pra1/F	ATCGGCATCCTCGCTCATTATG	pra1/R	TGCATGCTTGATCTCCGTTGCG	618
*pra2*	pra2/F	ACAGCATGTCTTCCCACCTTTTC	pra2/R	GACAAAGCAGCAGTGAACTGCC	606
*pra3*	pra3/F	CACAATTCCCATCACGGTGCTC	pra3/R	GAGCGAGAGCACTGATGGAAAG	582
*mfa1.2*	mfa1.2/F	CACAATGCCCTTTATCTGATGG	mfa1.2/R	GTTGTGGTCAGGCTAACTAGGG	389
*mfa2.1*	mfa2.1/F	CATCTCGAGTAACCCCTGAACA	mfa2.1/R	GAGATAATCAAAGGTGGGCGGG	498
*mfa3.2*	maf3.2/F	CAGTAGCACCTGTCTTCCTC	maf3.2/R	ACGCAAGTCGAGTAGCCGAGAG	331
*bW1*	bW1/F	CAAATCCAATCCATGGTAGCCG	bW1/R	CTGACAGCCGTAATCGAAGTTG	458
*bW2*	bW2/F	CAAGAATGTTCACGCCTTAGCC	bW2/R	CTTGTACCATTCGAGTGGATGC	452
*bW3*	bW3/F	CAGCACTTCTCCTCTTGTCGAG	bW3/R	TGATCAGCAGAGACATCGAGAG	480
*bE1*	bE1/F	CAACTCCTCGAACTACTCACTG	bE1/R	CGTCCAACTTGTGAGTCAGAAC	622
*bE2*	bE2/F	GGAACTAAGCAAGCCTTTTCGG	bE2/R	TCCAGTGCGGATAGGAATGTTG	627
*bE3*	bE3/F	ACCTTCTCCTTCGCCGATTTAC	bE3/R	TTCAGAACTGAGGGAAGAGGTC	632
ITS2/28S rRNA	UeITS2/F	AAGACGGACGAAAGCTTGAA	Ue28SrRNA/R	AATCCCAGGCCGCATCTCT	625

^1^ Primer sequences were adapted from Zhang *et al.* (2017) and Tu *et al.* (2019), except for those for pra2/F, maf3.2/F, and UeITS/F. The primers of pra2/F, maf3.2/F, and UeITS2/F were designed based on GenBank accession sequences KT343777.1, KT343769.1, and AB211904.1, respectively.

**Table 2.** Hyphal formation and mating types of strains isolated from the stem gall tissue of Ittenkou and Shirakawa cultivars

Cultivar	Strain	Presence of aerial hyphae	Mating type
Ittenkou (normal type)	I-1-(1)-A	–	MAT-2
	I-1-(2)-A	–	MAT-2
	I-1-(2)-C	–	MAT-2
	I-2-(3)-A	–	MAT-2
	I-2-(3)-E	–	MAT-2
	I-3-(3)-A	+	MAT-2, MAT-3
	I-3-(3)-B	+	MAT-2, MAT-3
	I-3-(3)-C	+	MAT-2, MAT-3
	I-4-(1)-A	+	MAT-2, MAT-3
	I-4-(1)-B	+	MAT-2, MAT-3
Shirakawa (normal type)	Sn-1-(1)-B1a	+	MAT-2, MAT-3
	Sn-1-(1)-B1b	+	MAT-2, MAT-3
	Sn-1-(1)-B1c	+	MAT-2, MAT-3
	Sn-1-(1)-B2a	+	MAT-2, MAT-3
	Sn-1-(1)-B2b	–	MAT-2
	Sn-1-(1)-F1	–	MAT-2
	Sn-1-(1)-F2	–	MAT-2
	Sn-1-(1)-F3	+	MAT-2, MAT-3
	Sn-1-(3)-C	–	MAT-2
	Sn-2-(2)-B	–	MAT-2
	Sn-2-(2)-D	–	MAT-2
	Sn-2-(2)-E	–	MAT-2
Shirakawa (delayed type)	Sd-2-(1)-A	–	MAT-2
	Sd-2-(1)-F	–	MAT-2
	Sd-3-(1)-B	–	MAT-2
	Sd-3-(1)-D1	–	MAT-2
	Sd-3-(1)-D2	–	MAT-2
	Sd-3-(1)-D3	–	MAT-2

**Table 3.** Mating types of *Ustilago esculenta* strains with retarded sporidial growth

Strain	Maximum diameter^1^ (visual observation)			Sporidial growth confirmed with a stereomicroscope^2^		Mating type
	PDA	YEPS		PDA	YEPS	
A12	2‍ ‍mm	2‍ ‍mm		n.d.	n.d.	MAT-2
A13	2‍ ‍mm	invisible		n.d.	+	MAT-2
α1	1‍ ‍mm	invisible		n.d.	–	MAT-3
α20	Invisible	invisible		+	–	MAT-2, MAT-3
α25	Invisible	invisible		+	+	MAT-2, MAT-3
β3	Invisible	invisible		+	+	MAT-2, MAT-3
β8	1‍ ‍mm	invisible		n.d.	+	MAT-2
β9	1‍ ‍mm	invisible		n.d.	+	MAT-2
β11	2‍ ‍mm	0.5‍ ‍mm		n.d.	n.d.	MAT-2
γ17	Invisible	invisible		–	+	MAT-2, MAT-3

^1^ The maximum diameter of visually observable colonies was measured roughly with a ruler. ^2^ The presence (+) or absence (–) of sporidial growth was confirmed by observations under a stereomicroscope. n.d.=not determined.

**Table 4.** Mating types of *Ustilago esculenta* strains from different production areas in Japan and China

MAFF ID	Strain	Production area (city)	Mating type
241286	TY64	Nagano Pref. (Nagano)	MAT-2
241684	TY65	Nagano Pref. (Nagano)	MAT-2
305616	yeast4	China	MAT-2, MAT-3
305618	MC-2	Mie Pref.	MAT-2
305619	MJ-2	Shiga Pref.	MAT-1
305620	IF-6	Okinawa Pref.	MAT-2, MAT-3
305621	ON-3	Okinawa Pref.	MAT-2
327436	J1	Ibaraki Pref. (Tsuchiura)	MAT-2, MAT-3
327440	J5	Ibaraki Pref. (Tsuchiura)	MAT-2
327443	J8	Ibaraki Pref. (Tsuchiura)	MAT-2, MAT-3
