# Supplementary material for: Mating Types of Ustilago esculenta Infecting Zizania latifolia Cultivars in Japan Are Biased towards MAT-2 and MAT-3
Source: Microbes Environ. 2023 Sep 14;38(3):ME23034. doi: 10.1264/jsme2.ME23034 (PMC10522849; doi:10.1264/jsme2.ME23034)
Supplement: Supplementary file 1 — Supplementary Material [file 38_23034_s1.pdf]

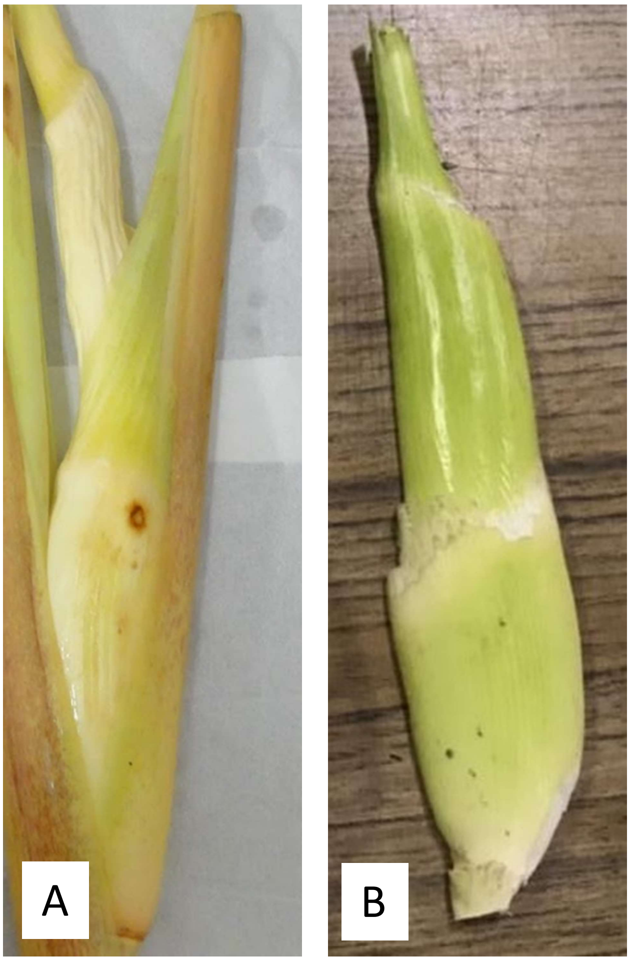

Fig. S1. Makomotake stem galls of *Zizania latifolia* cultivars.
A: Ittenkou, B: Shirakawa.

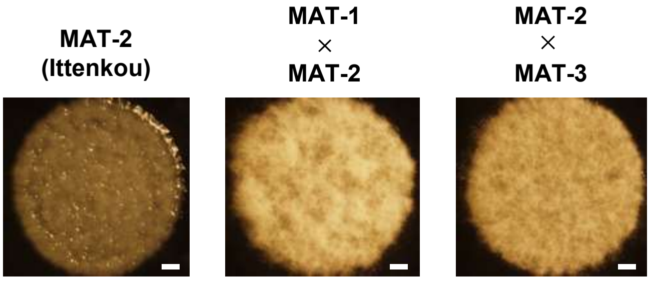

Fig. S2. Morphology of colonies formed by *in vitro* mating experiment using MAT-1 and MAT-3 strains from Shirakawa and a MAT-2 strain from Ittenkou.
Sporidia of a MAT-2 strain from Ittenkou or a combination of two strains (MAT-1 × MAT-2 and MAT-2 × MAT-3) were grown on YEPS at 28˚C. Images were captured after 14-day incubation. MAT-1 = strain MJ-2; MAT-2 = strain In-2-(3)-A; MAT-3 = strain α1. Bar = 1 mm.

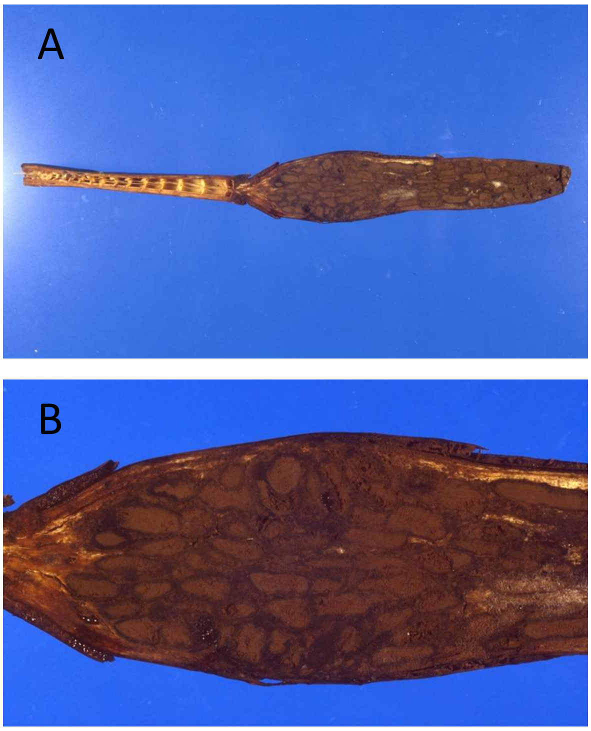

Fig. S3. A stem gall of a *Zizania latifolia* plant infected with *Ustilago esculenta*, collected from Shiga Prefecture in Japan. A. The photograph of a brown-blackish gall infected with *U. esculenta* showing stem tissue full of sori. B. The basal part of the gall connecting the culm is enlarged.
